# The Genetic Intersection of Neurodevelopmental Disorders and Shared Medical Comorbidities – Relations that Translate from Bench to Bedside

**DOI:** 10.3389/fpsyt.2016.00142

**Published:** 2016-08-22

**Authors:** Jasmine T. Plummer, Alexis J. Gordon, Pat Levitt

**Affiliations:** ^1^Institute for the Developing Mind, The Saban Research Institute, Children’s Hospital Los Angeles, Los Angeles, CA, USA; ^2^Keck School of Medicine, University of Southern California, Los Angeles, CA, USA; ^3^Department of Pediatrics, Keck School of Medicine, University of Southern California, Los Angeles, CA, USA

**Keywords:** mental illness, neurogenetics, autism, schizophrenia, brain development

## Abstract

Most psychiatric disorders are considered neurodevelopmental, and the associated genes often are expressed in tissues outside of the brain. This suggests a biological relatedness with medical co-occurrences that could have broad clinical implications for diagnosis and patient management over a lifetime. A qualitative integration of public data from genetic consortia of psychiatric disorders and medical comorbidities explores the question of whether genetically associated psychiatric illnesses present with co-occurring disturbances can be used to define specific mental–physical health relations. Novel patterns of gene-disorder relations appear with approximately one-third of conservatively defined, consortia-generated candidate risk genes with multiple psychiatric diagnoses. Moreover, nearly as many genes overlap with non-psychiatric phenotypes, including cardiovascular, renal, respiratory, and metabolic disturbances. While the landscape of genetic risk will change as study populations are expanded and biological confirmations accrue, the current relationships suggest that a mostly siloed perspective of gene relatedness to one categorical psychiatric diagnosis is not clinically useful. The future holds the promise that once candidates are fully validated, genome screening and mutation identification will bring more precision for predicting the risk for complex health conditions. Our view is that as genetic data are refined, continuing to decipher a shared pattern of genetic risk for brain and peripheral organ pathophysiology is not simply an academic exercise. Rather, determining relatedness will impact predictions of multifaceted health risks, patient treatment, and management.

## Introduction

Neurodevelopmental disorders (NDDs) are caused by abnormalities of brain development due to somatic or germ line mutations, and include certain psychiatric disorders (1). NDDs affect an estimated 15% of the population and are a major source of economic and clinical care burden on the healthcare system (National Institute of Mental Health Statistics).^1^ NDDs, such as schizophrenia (SCZ), autism spectrum disorder (ASD), attention-deficit hyperactive disorder (ADHD), and mood disorders [bipolar disorder (BD) and major depressive disorder (MDD)], are characterized by a range of brain-based symptoms, some of which overlap between diagnostic categories (2). The de-emphasis on specific clinical categories and a greater focus on dimensions of behavioral and neurobiological measures reflects a goal of using new discoveries to inform treatment that extend beyond classic disease boundaries (3). Yet, the origins of symptoms overlap and clinical variation remains a challenge to discern. The pathophysiological mechanisms of NDD symptoms are complex and the developmental mechanisms that underlie psychiatric disorder risk remain largely unknown. Advances in the understanding of altered gene expression and its role in NDD risk are providing a more advanced framework for deciphering disease etiologies.

The co-occurrence of non-psychiatric and mental health disturbances may not be surprising, given that nearly all genes in the genome are expressed in the developing human brain, with subsets found in peripheral organs. There is increasing interest in studies of the relations between specific NDDs, such as SCZ and BD, and enrichment of medical conditions, including cardiovascular disease, diabetes, and respiratory conditions (4). The question then arises: given the current state of mental illness and medical genetic discoveries, how much shared genetic risk exists between the brain and other organs? If, for example, coding mutations impact NDD risk gene function in the brain, is it likely that other organ systems in which the gene functions will also be negatively impacted? NDD gene expression may be more complex when allelic variations occur in the regulatory regions of these genes because the same gene may be regulated quite differently in distinct tissues. While not yet understood in terms of impact on specific disorder symptoms, recent analyses of polymorphisms from genome-wide association studies (GWAS) have demonstrated significant genetic relations across certain disorders, such as between SCZ with BD (5) and with anorexia nervosa (5). We have conceptualized these potential relations by analyzing current gene–disease findings across physical and mental illnesses to better understand co-occurring clinical patterns.

Given that different psychiatric genetic consortia have reported “high confidence” risk and causal genes, we posited that analysis of these genes might promote a refined understanding of the genetic origins of and relations to non-psychiatric comorbidities. This perspective is put forth with the understanding that the landscape of genetic risk is certain to change over time due to methodological improvements in genetic discovery and analysis, biological validation of risk polymorphisms, and continued increase of subjects analyzed. The analysis revealed relationships between consortia-defined NDD risk genes and medical comorbidities.

## A Current NDD Gene List: A Starting Point but Ever-Evolving

In the analysis, the designation of NDD included syndromic disorders (SYN – categorized as genetically determined Mendelian NDDs, such as Rett Syndrome or Fragile-X Syndrome), as well as typically childhood (ASD and ADHD) or adult-onset (BD, MDD, and SCZ) brain-based disturbances (Table 1). A NDD risk gene list was compiled by selecting “high confidence” risk or causal genes, as defined by each consortium, for a specific categorical diagnosis using criteria established by the following psychiatric disorder databases:

(1) ADHD: the “Hot Gene List” from ADHDgene.^2^
(2) ASD: genes listed as Category 1 (high confidence) and Category 2 (strong candidate) on Simons Foundation Autism Research Initiative Gene (SFARIgene).^3^
(3) BD: the “Core Gene List” from BDgene.^4^
(4) MDD: the top 20 gene associations with MDD from PsyGeNet.^5^
(5) SCZ: because the SCZ genetic database (SCZgene) had not been updated since 2009, we used data generated by the 2014 Schizophrenia Working Group of the Psychiatric Genomics Consortium (6). This is the most extensive psychiatric genetic study to date. However, all data meeting genome-wide significance were not included, because many polymorphisms are not within close proximity to coding genes. Thus, we included only genes from loci that had single gene hits (54 of 108), recognizing that non-coding RNA species and regulatory polymorphisms are not included.
(6) SYN: genes listed in Category S (syndromic) of SFARIgene.^3^

**Table 1:** **Neurodevelopmental (NDD) and OMIM-defined physical disorders**.

Disorder	Abbreviation and Neurodevelopmental Disorder Color Code
Schizophrenia	SCZ
Bipolar disorder	BP
Syndromic disorders	SYN
Autism spectrum disorder	ASD
Attention-deficit hyperactivity disorder	ADHD
Major monopolar disorder	MDD
Intellectual disability	ID
Craniofacial	CRANIO
Epilepsy	EPI
Musculoskeletal	MSK
Cardiovascular	CARDIAC
Ataxia/motor	MOTOR
Genitourinary	GU
Endocrine	ENDO
Cancer	CANCER
Ophthalmological	OPHTHO
Respiratory	RESP
Speech-language	SPEECH
Ear, nose, and throat	ENT
Gastrointestinal	GI
Dermatological	DERM
Allergy/immunological	IMMUNO

*Color codes demarcate genes in each specific NDD category*.

After compiling the NDD gene list, non-psychiatric phenotypes associated with each gene, generated from Online Mendelian Inheritance in Man (OMIM),^6^ were assigned using Biomart [ENSEMBL Genes 82, Homo sapiens gene (GRCh38.p3)]. The NDD gene list was uploaded by Entrez Gene IDs and then filtered for attributes by MIM Morbid Gene Description (e.g., phenotype associated in OMIM) (Ensembl Biomart).^7^ Note that there currently is debate in the literature on the voracity of identifying risk genes (7, 8). Our list includes genes that underlie highly penetrant, rare events that are thought to be causal, as well as genes associated with statistically significant polymorphisms in smaller cohorts with a categorical diagnosis, or larger patient cohorts of a certain diagnosis. The list should not be considered absolute, as current data do not typically include determination of the functional impact of genome-wide polymorphisms on specific genes. There currently are few examples of such studies (9–11).

Genes were first stratified by their psychiatric phenotype (ADHD, ASD, BD, MDD, SCZ, and SYN) (Table 1). Secondary categorization of NDD genes by non-psychiatric phenotypic comorbidities was done by separating genes into basic organ systems. These categories were (1) ears, nose, and throat (ENT – included tonsils, snoring, and hearing), (2) ophthalmologic (OPHTHO), (3) cardiac (CARDIAC), (4) respiratory (RESP), (5) gastrointestinal (GI), (6) genitourinary (GU), (7) endocrine (ENDO – included temperature regulation), (8) allergy/immunological (IMMUNO), (9) musculoskeletal (MSK), (10) dermatological (DERM), (11) craniofacial (CRANIO), and (12) neurological. Because these genes were associated with NDDs and, therefore, have many brain-associated disturbances, the neurological category was further subdivided into (1) epilepsy/seizures (EPI), (2) intellectual disability (ID), (3) speech–language impairments (SPEECH), and (4) ataxia/motor impairments (MOTOR). Including CANCER, there were 16 non-psychiatric categories (Table 1).

The non-psychiatric categories were not mutually exclusive, as comorbidities were often spectral. For example, genes associated with speech, language, and speech-related communication deficits were part of the same phenotypic SPEECH category. In addition, some genes were associated with communication delays secondary to ID. These were classified as ID and not the SPEECH category unless the genes were also associated with risk for specific SPEECH deficits. To highlight how a NDD gene was categorized in this analysis, we use transcription factor 4 (*TCF4*) as an example. *TCF4* had associations with ASD, BD, SCZ, and Pitt–Hopkins Syndrome (SYN), the latter causing clinical symptoms that include seizures, constipation, and myopia. The clinical disturbances associated with *TCF4* were assigned to the following non-psychiatric categorizations: (1) EPI, (2) GI, and (3) OPHTHO. Furthermore, *TCF4* had an association with Diabetes Mellitus II; accordingly, a categorization of endocrine disorders (ENDO) was included.

## NDD Risk Gene Relationships

**Figure 1:**
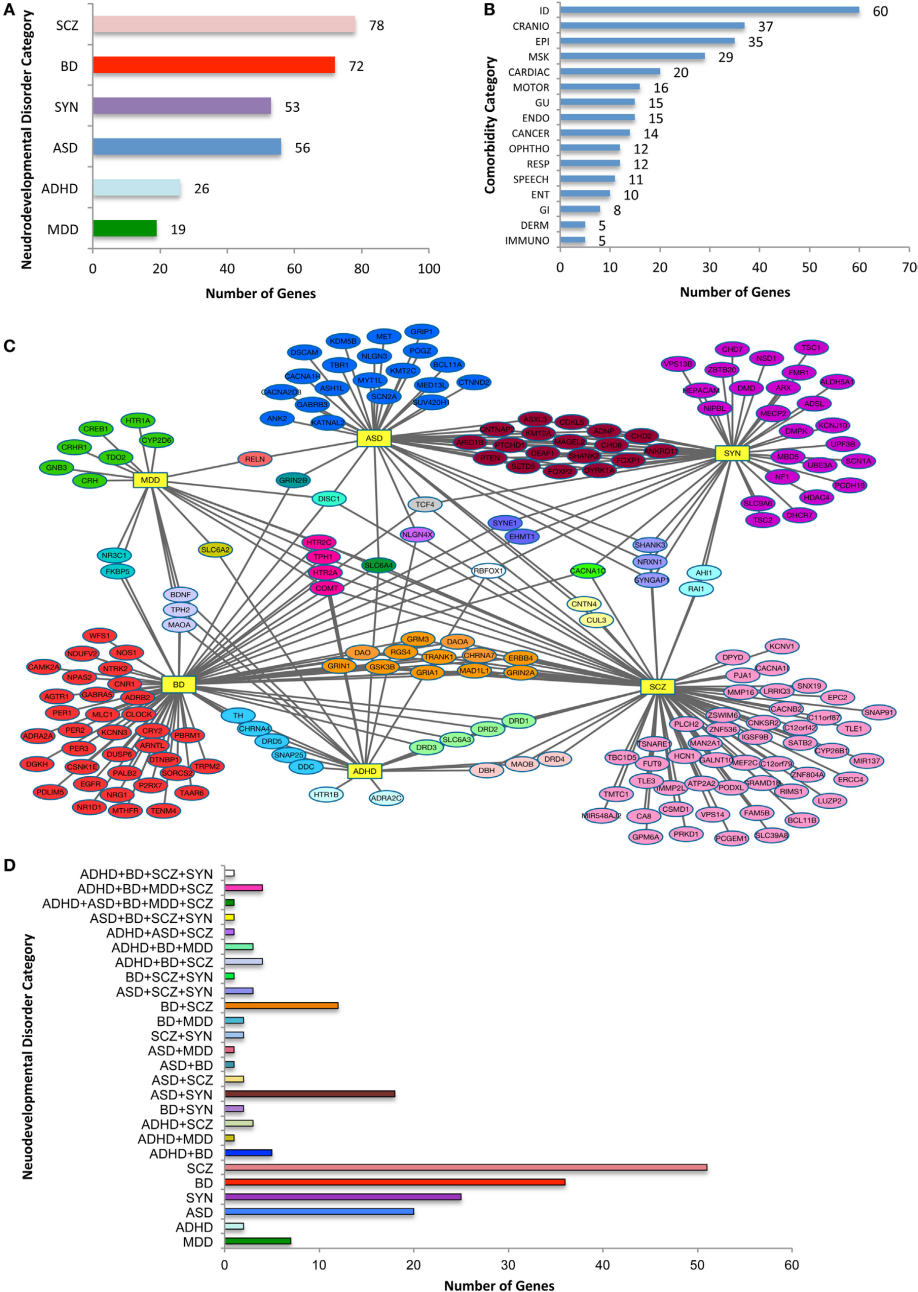
**Categorization of neurodevelopmental disorder (NDD) risk genes and their associated comorbidities**. **(A)** NDD categories and the number risk genes for attention-deficit hyperactive disorder (ADHD), autism spectrum disorder (ASD), bipolar disorder (BD), major depressive disorder (MDD), schizophrenia (SCZ), and syndromic neurodevelopmental disorders (SYN). Note that because a single gene may be assigned to more than one category, the total is greater than the 208 genes analyzed. **(B)** The distribution of NDD risk genes with OMIM-generated, related non-psychiatric conditions. The phenotypic categories listed in **(A,B)** are not mutually exclusive. **(C)** Diagram of relationship between NDD risk genes. NDD risk genes depicted with their primary psychiatric associations. Abbreviations for each NDD are as noted for **(A)**, and are shown as hubs (yellow rectangles). Note the connection of NDD genes associated with one or more psychiatric conditions. **(D)** Histogram with color code that depicts NDD risk genes associated with a single or two or more psychiatric categories, the latter comprising nearly half of the 208 psychiatric NDD genes. NDD abbreviations are noted in Table 1. *X*-axis indicates number of genes in each combination or single category.

There are 208 individual risk genes on the selection list (Figure 1). There were substantial multiple category diagnoses for ~50% of the genes, with 78 associated with SCZ, 26 genes with ADHD, 49 with ASD, 72 with BD, 19 with MDD, and 53 with SYN (a total greater than 208 individual risk genes). Note that the data are relatively skewed, because of more available information from the large, GWAS SCZ studies compared to the other five psychiatric diagnoses, yet there was considerable overlap with other diagnostic categories. Across the 16 non-psychiatric phenotypic categories, there was a broad distribution of associated NDD risk genes (Figure 1B). Not surprisingly, the largest number of NDD risk genes was associated with ID, with the other brain-based phenotypes also highly represented. Consistent with the notion that NDD risk genes likely affect organ systems other than the brain, approximately half of the NDD risk genes were associated with non-brain-based disturbances. The largest of these phenotypic categories included CRANIO, MSK, CARDIAC, GU, and ENDO disturbances. Thus, in addition to non-psychiatric brain-based abnormalities, NDD genes have considerable connections to disturbances in other tissues and physiological targets, an observation not typically considered when characterizing NDDs.

**Figure 2:**
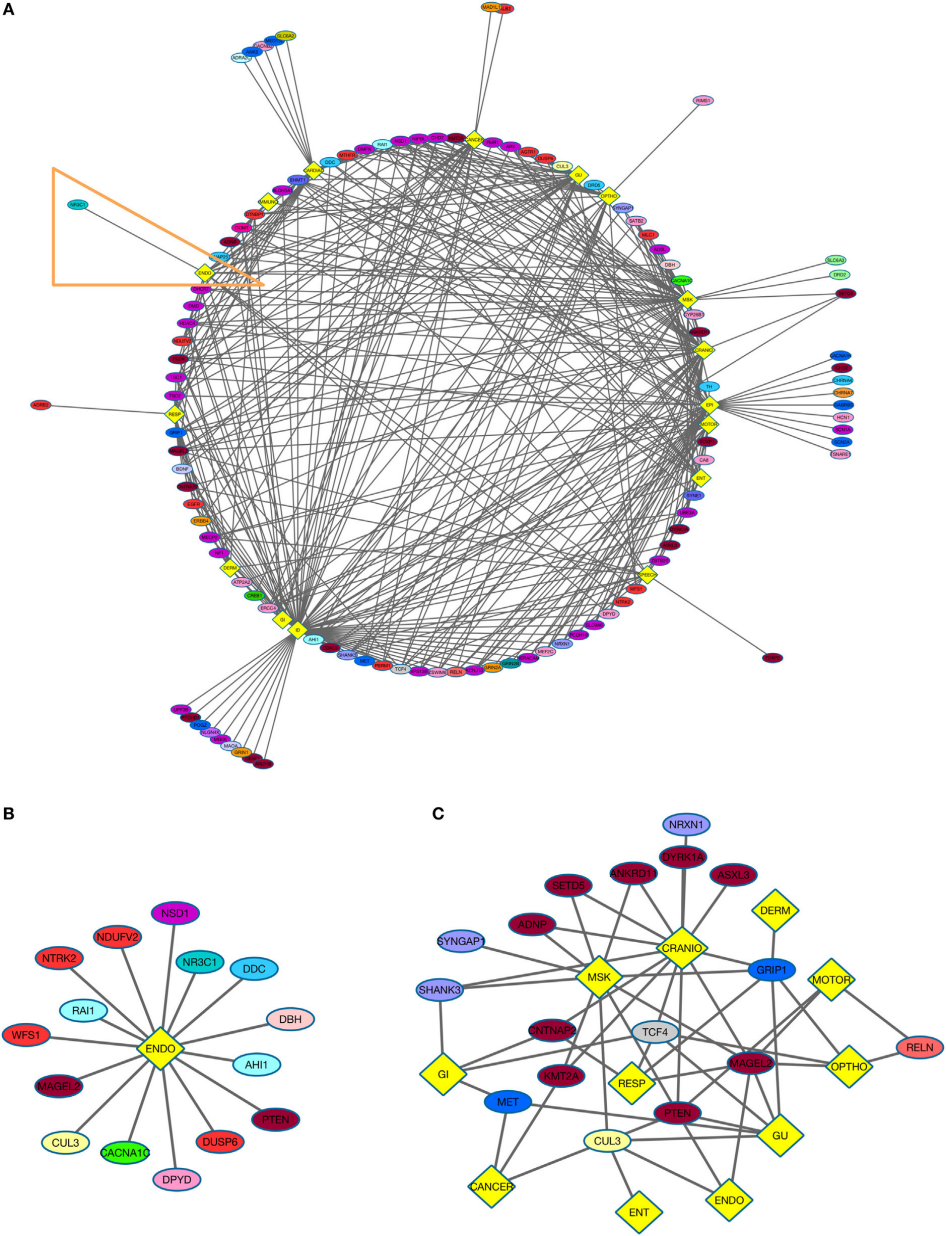
**NDD risk genes and their non-psychiatric conditions**. **(A)** Diagram displays NDD risk genes (ellipses) and their associated non-psychiatric conditions (yellow diamonds). **(B)** NDD risk genes and their association with endocrine disorders (ENDO). This group is derived from Figure 2A (orange outline). **(C)** Autism spectrum disorder (ASD) comorbidity subgroup. The analysis depicts relations of genes associated with ASD and other psychiatric disorders with medical conditions (yellow diamonds). See Table 1 for color code of genes associated with each NDD, and Figure 1D for the color code for various combinations of NDDs to which a gene was assigned.

Analysis of NDD genes and their psychiatric phenotypes revealed a striking degree of overlap between seemingly unrelated disorders (Figure 1C). Overall, 33% of the 208 genes were associated with two or more disorders. For instance, of the 49 ASD-related risk genes, 28 were noted with one additional NDD (Figure 2B), with the largest subgroup (18 genes) shared by ASD and SYN. By contrast, only two genes were shared by ASD and SCZ alone. When considering the 72 BD risk genes on the list, interesting subgroups of shared genes emerged. Specifically, discrete subgroups were associated with BD and ADHD (5 genes), BD and SCZ (12 genes), and BD and MDD (2 genes). Intriguingly, genes associated with these subgroups encoded proteins involved in diverse cellular functions. For example, BD + SCZ subgroup included a d-amino acid oxidase (DAO) and its activator (DAOA), a Wnt pathway enzyme (GSK3β), a mitotic protein (MAD1L1), an ATP binding protein (TRANK1), a cell signaling receptor tyrosine kinase (ERRB4), a regulator of G protein signaling (RGS4), a cholinergic receptor (CHRNA7), and glutamate receptors (GRM3, GRIN1, and GRIA1). This is distinct from the BD + MDD subgroup, which included a protein-modifying enzyme (FKBP5) and a nuclear receptor (NR3C1). There typically was limited overlap of gene function between subgroups associated with BD (Figure 1D). Finally, there was a modest number of genes in three or more NDD categories. This is best exemplified by SLC6A4, which was associated with all of our NDD categories, with the exception of SYN. This is consistent with the idea that perturbations of the same genetic pathway can result in different psychiatric diagnoses, likely due to genetic and environmental phenotypic modifiers.

Neurodevelopmental disorder risk genes across all six psychiatric categories shared common non-psychiatric comorbidities (Figure 2A) To gain insight into the relatedness of psychiatric categories and non-psychiatric comorbidities, two groups, both of which included genes from the six NDD categories, were created. The ENDO group comprises the following: SCZ (1 gene), ASD (3), ADHD (1), MDD (2), BD (3), and SYN (5) (Figure 2B). NDD genes from all six psychiatric categories were present in the ENDO subgroup, demonstrating the likelihood of non-neurological comorbidities related to specific NDDs. For example, the NDD risk gene PTEN is observed in the ENDO subgroup but also is associated with several other non-neurological comorbidities (CARDIAC, GI, GU, MSK, CRANIO, and CANCER).

Finally, we explored the comorbidities associated with a specific NDD by generating an ASD risk gene subgroup (Figure 2C). We focused on non-neurological associations, excluding ID and EPI. Remarkably, there were 11 organ system categories associated with ASD risk genes. Certain genes were also associated with multiple non-neurological comorbidities. For example, MAGEL2 was associated with seven organ systems comorbidities: MSK, GU, ENDO, OPHTHO, CRANIO, MOTOR, and RESP. Within this subgroup, the genes associated with MSK also exhibited shared risk for other disorders (including SCZ and SYN). Certain subgroups of genes associated with different NDDs were related to similar phenotypic categories. For instance, CNTNAP2 (ASD + SYN) and TCF4 (ASD + BD + SYN + SCZ) were associated with CRANIO, GI and RESP. Shared ASD risk genes also overlap in their relations to non-neurological phenotypes.

These relationships highlight that there are many non-neurological comorbidities associated with the NDD risk genes. Thus, as medical genetics evolves, so does the necessity to reduce single gene-single disease boundaries and view a broader pathophysiological impact on other organ systems. The analyses presented here may help reframe how clinicians and scientists approach the treatment and study of NDDs beyond single conditions. This perspective aligns with the recent Research Domain Criteria (RDoC) to de-emphasize categories of diagnoses, and instead utilize functional dimensions to delineate and ultimately treat clinical symptoms (3, 12). It is important to emphasize that the present study does not address the relative strength of the relations nor the specific genetic variants that drive the relations, although recent investigations are attempting to address this (5, 7). Additionally, it is important to emphasize that unknown referral and ascertainment bias in the NDD populations that dominate some of the genetic consortia may provide a biased sample with unknown or absent major medical comorbidities. Ultimately, a population study will be needed to assess medical comorbidities, such as the AGRE and Simons Simplex collections for the studies of ASD. As new data from deep gene sequencing and larger population association analyses accumulate, additional analyses will be capable of bringing even more clarity to novel patterns of relations across psychiatric NDDs and medical conditions.

## Advancing A Shared Biology for Future Investigations

We focused on gaining a perspective on the degree to which there are relationships between NDDs and non-psychiatric comorbidities, through either common or rare genetic risk as the field currently defines these. The relationships reported here highlight a potentially shared biology for genes expressed during brain and peripheral organ system development. It will be important in future studies to align a refined genetic understanding with comparative analyses of gene expression for specific NDDs and medical conditions. Because genes encode proteins that are mediating different functions in distinct cell and developmental contexts, gene mis-regulation or dysfunction due to allelic variation or coding mutations may produce unique, context-specific outcomes. These genetic perturbations could contribute to different phenotypes observed in psychiatric disorders and other non-psychiatric disturbances. That is, the same gene can be associated with seemingly unrelated disorders and conditions because the gene product may be part of different molecular networks in distinct tissue and cell settings. In addition, clinical heterogeneity, even within a single-gene syndrome, such as Rett or Angelman Syndromes, is substantial. The combination of clinical heterogeneity and diversity of biological functions creates challenges for understanding the contribution of specific genes to the pathogenesis and pathophysiology of a specific categorical disorder. Nonetheless, the relations between non-psychiatric disturbances, mental illness, and NDD risk genes provide an important framework for recognizing the clinical complexity of any NDD. Currently, standard care focuses on the neurological aspect of psychiatric NDDs or mental illness. Here, we suggest a potential platform for utilizing a more holistic method for treating other organ systems along with NDDs.

### Highlighting Autism Comorbidities

Syndromic Disorder genes were the largest subgroup of overlapping ASD risk genes. Since disruptions in SYN NDDs by definition affect multiple organ systems, it was not surprising that there were numerous co-occurring conditions associated with ASD genes, including GI, CANCER, IMMUNO, GU, and CARDIAC disorders. GI was composed of eight genes: CDKL5, CHD7, CNTNAP2, DHCR7, MET, NIPBL, PTEN, and TCF4. This coincides with the high prevalence of GI disturbances, over 40%, in children with ASD (13–15), and is consistent with the perspective that functional genetic variations that increase risk for ASD may also underlie ASD-associated GI dysfunctions. For example, the MET receptor regulates enteric neuron development and intestinal motility (16), and in humans, *MET* promoter variant rs1858830, a common functional promoter polymorphism that increases the risk for ASD, is enriched further in individuals with co-occurring GI dysfunction (13). Interestingly, genes within the ASD-GI grouping differ in their protein function, consistent with the idea that different cellular mechanisms may underlie similar non-psychiatric comorbidities, as well as other associated ASD comorbidities. For example, in contrast to MET, CDKL5, a transcription factor, is not only associated with ASD risk but also is a susceptibility locus for early infantile epileptic encephalopathy 2 (EIEE2). In addition to presenting with seizures, children with EIEE2 present with GI disturbances, subtle dysmorphic facial features, sleep disturbances, and stereotypic hand movements (15).

## Informing Treatment and Patient Management

Viewing non-psychiatric comorbidities of NDDs through a current understanding of genetic relations raises a number of interesting possibilities. This perspective promotes the idea of a more integrated approach to treatments of individuals with a particular diagnosis, more predictable treatment responses and better predictive side effect profiles. In fact, research relevant to this approach is presently underway (17). For example, several genes are associated with altered enzyme levels that can impact drug metabolism (18). Additionally, certain medications used to treat specific NDDs also are associated with metabolic syndrome or an increased risk for cardiovascular disease (19). Ultimately, as the identification of high confidence risk genes undergoes modification with new human genetic discoveries and biological validation, identifying non-psychiatric conditions associated with NDD genes may be useful as a practical screen for recognizing possible treatment side effects and promoting the careful tailored treatments that avoid deleterious effects. These connections also may be useful for interpreting potential health implications based on clinical genetic assays that are being implemented more routinely.

There may be additional benefits in a greater understanding of the relation between co-occurring non-psychiatric and psychiatric disturbances. First, the stigma and misunderstanding that accompany the diagnosis of NDDs or psychiatric disorders, in contrast to medical conditions, remain significant, but a better understanding of genetic relations may advance society’s viewpoints (20). We suggest that an increased recognition of co-occurring psychiatric and non-psychiatric disorders, and the genetic and biological factors that underlie co-occurrences, may reframe opinions on causality. This reframing may help alter the view that individual weakness is a risk for psychiatric disorders and instead refocus society’s viewpoint toward the contribution of genetic risk for mental illness. Deciphering the patterns of non-psychiatric disturbances associated with specific NDDs and mental illnesses also will lead to a better understanding of the bio-psycho-social complexity of challenges faced by these individuals and their families (21). Second, there are limited efforts to develop integrated preclinical research models that incorporate genetic strategies for both non-psychiatric and psychiatric disturbances. There is, thus, a significant knowledge gap, due to the rising understanding that peripheral organ systems, such as the GI or IMMUNO system, can directly influence brain development, structure, and function (22). Future clinical and basic investigations into the shared genetic relations among psychiatric and peripheral organ function will provide opportunities for both healthcare providers and researchers to build a framework to address the challenges of complex patient care and disorder etiologies.

## Author Contributions

JP and PL conceived of the project. JP developed the analysis strategy. JP and AG contributed to the analysis and wrote the first draft of the manuscript. PL supervised the project, and contributed to revisions of the data analyses and writing of the manuscript. All authors discussed the results and implications and commented on the manuscript at all stages.

## Conflict of Interest Statement

The authors declare that the research was conducted in the absence of any commercial or financial relationships that could be construed as a potential conflict of interest.

## Footnotes

^1^http://www.nimh.nih.gov/health/statistics/index.shtml

^2^http://adhd.psych.ac.cn/topGene.do

^3^https://gene.sfari.org/autdb/GS_Home.do

^4^http://bdgene.psych.ac.cn/topGene.do

^5^http://www.psygenet.org/

^6^http://www.omim.org

^7^http://uswest.ensembl.org/biomart/martview/5c4ed4a0df8a4edd185363c07c92e0ce
