# Erratum: The Genetic Intersection of Neurodevelopmental Disorders and Shared Medical Comorbidities – Relations that Translate from Bench to Bedside

**DOI:** 10.3389/fpsyt.2016.00166

**Published:** 2016-09-30

**Authors:** Frontiers Production Office

**Affiliations:** ^1^Frontiers Production Office, Frontiers, Lausanne, Switzerland

**Keywords:** mental illness, neurogenetics, autism, schizophrenia, brain development

**Reason for erratum:**

Due to a typesetting error, some information went missing on the *y*-axis of Figure 1B in the original publication.

**Figure 1:**
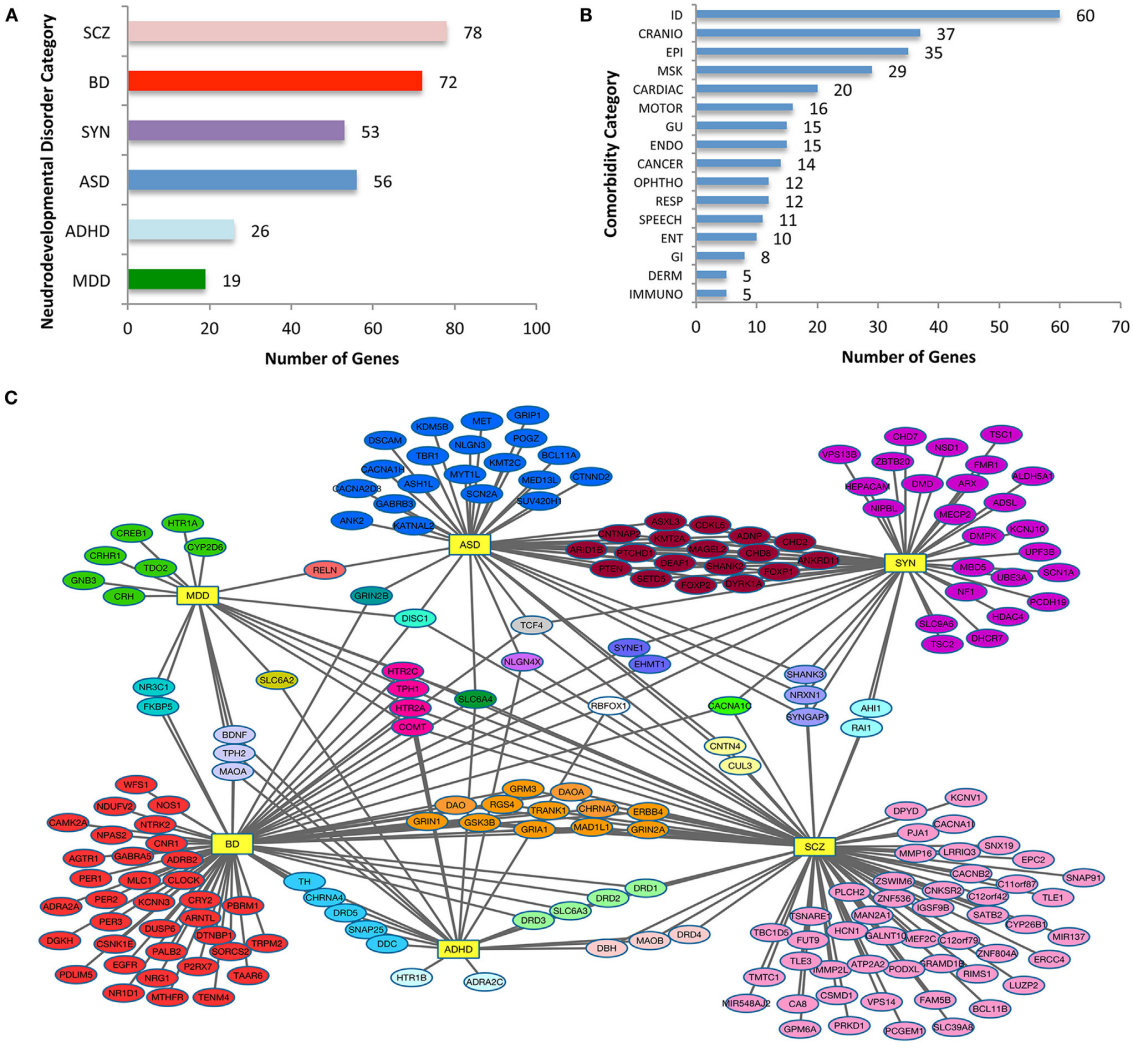

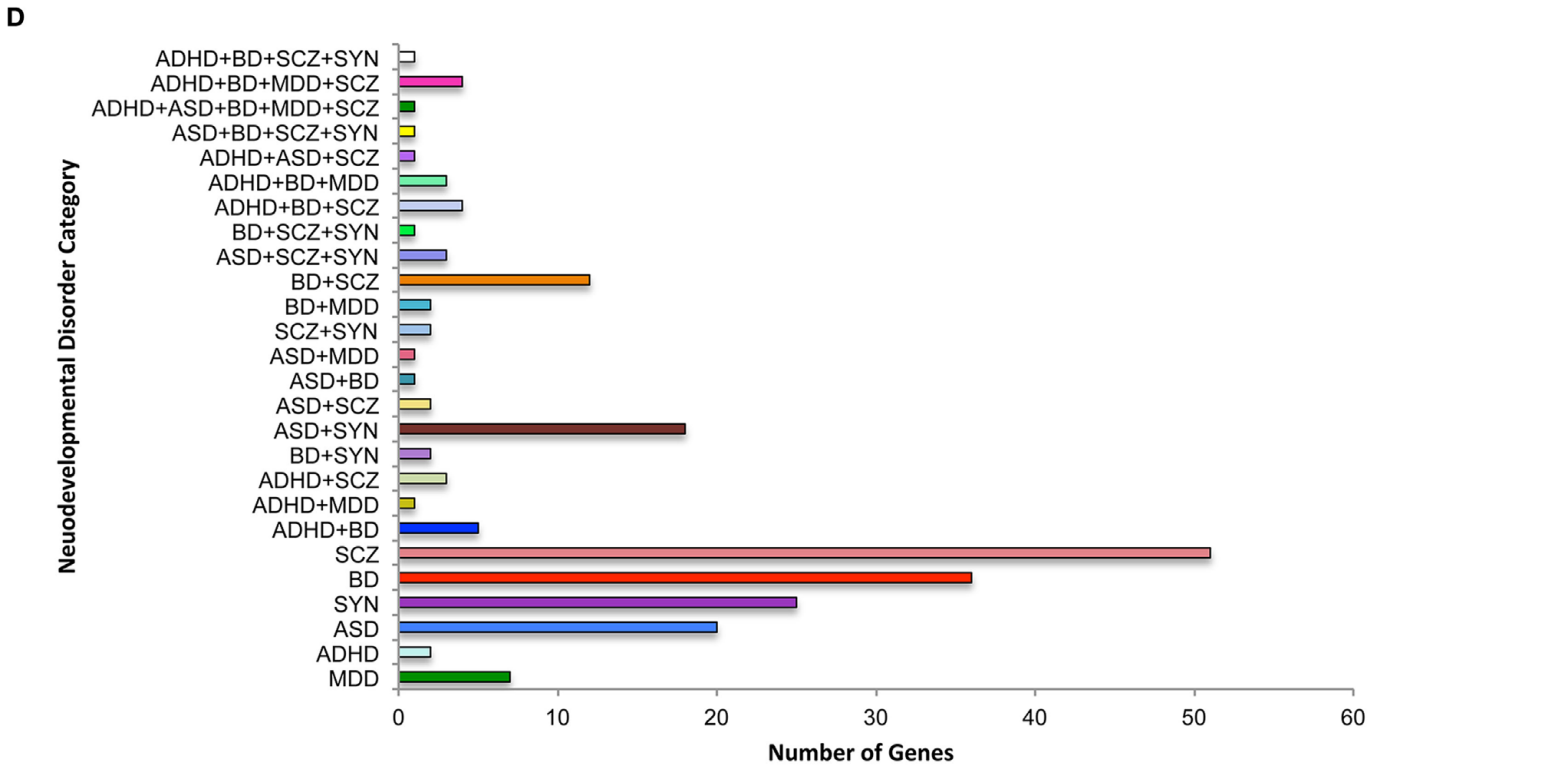
**Categorization of neurodevelopmental disorder (NDD) risk genes and their associated comorbidities**. **(A)** NDD categories and the number risk genes for attention-deficit hyperactive disorder (ADHD), autism spectrum disorder (ASD), bipolar disorder (BD), major depressive disorder (MDD), schizophrenia (SCZ), and syndromic neurodevelopmental disorders (SYN). Note that because a single gene may be assigned to more than one category, the total is greater than the 208 genes analyzed. **(B)** The distribution of NDD risk genes with OMIM-generated, related non-psychiatric conditions. The phenotypic categories listed in **(A,B)** are not mutually exclusive. **(C)** Diagram of relationship between NDD risk genes. NDD risk genes depicted with their primary psychiatric associations. Abbreviations for each NDD are as noted for **(A)**, and are shown as hubs (yellow rectangles). Note the connection of NDD genes associated with one or more psychiatric conditions. **(D)** Histogram with color code that depicts NDD risk genes associated with a single or two or more psychiatric categories, the latter comprising nearly half of the 208 psychiatric NDD genes. NDD abbreviations are noted in Table 1. *X*-axis indicates number of genes in each combination or single category.

The publisher apologizes for this error, and the correct version of Figure 1 appears below. This error does not change the scientific conclusions of the article in any way.

The original article has been updated.

