# Supplementary material for: The Genetic Intersection of Neurodevelopmental Disorders and Shared Medical Comorbidities – Relations that Translate from Bench to Bedside
Source: Front Psychiatry. 2016 Aug 22;7:142. doi: 10.3389/fpsyt.2016.00142 (PMC4992686; doi:10.3389/fpsyt.2016.00142)
Supplement: Supplementary file 1 [file table_1.docx]

Supplemental Table 1 – List of high confidence risk genes compiled from NDD genetic consortia

| **RISK GENES** | | | | | |
| --- | --- | --- | --- | --- | --- |
| ADNP | CHRNA7 | EHMT1 | KCNN3 | NSD1 | SNAP25 |
| ADRA2A | CLOCK | EN2 | KCNV1 | NTRK2 | SNAP91 |
| ADRA2C | CNR1 | EPC2 | KDM5B | OXTR | SNX19 |
| ADRB2 | CNTN4 | ERBB4 | KMT2A | P2RX7 | SORCS2 |
| ADSL | CNTNAP2 | FAM5B | KMT2C | PALB2 | SUV420H1 |
| AGTR1 | COMT | FKBP5 | LRRIQ3 | PBRM1 | SYNE1 |
| AHI1 | CREB1 | FMR1 | LUZP2 | PCDH19 | SYNGAP1 |
| ALDH5A1 | CRH | FOXP1 | MAD1L1 | PCGEM1 | TAAR6 |
| ANK2 | CRHR1 | FOXP2 | MAGEL2 | PDLIM5 | TBC1D5 |
| ANKRD11 | CRY2 | FUT9 | MAN2A1 | PER1 | TBR1 |
| ARID1B | CSMD1 | GABRA5 | MAOA | PER2 | TCF4 |
| ARNTL | CSNK1E | GABRB3 | MAOB | PER3 | TDO2 |
| ARX | CTNND2 | GALNT10 | MBD5 | PJA1 | TENM4 |
| ASH1L | CUL3 | GNB3 | MECP2 | PODXL | TH |
| ASXL3 | DAO | GPM6A | MED13L | POGZ | TLE1 |
| ATP2A2 | DAOA | GRAMD1B | MEF2C | PRKD1 | TLE3 |
| AVPR1A | DBH | GRIA1 | MET | PTCHD1 | TMTC1 |
| BCL11A | DDC | GRIK2 | MIR137 | PTEN | TPH1 |
| BCL11B | DEAF1 | GRIN1 | MIR548AJ2 | RAI1 | TPH2 |
| BDNF | DGKH | GRIN2A | MLC1 | RBFOX1 | TRANK1 |
| C11orf87 | DHCR7 | GRIN2B | MMP16 | RELN | TRPM2 |
| C12orf42 | DISC1 | GRIP1 | MSNP1AS | RGS4 | TSC1 |
| C12orf79 | DMD | GRM3 | MTHFR | RIMS1 | TSC2 |
| CA8 | DMPK | GSK3B | MYT1L | SATB2 | TSNARE1 |
| CACNA1C | DPYD | HDAC4 | NDUFV2 | SCN1A | UBE3A |
| CACNA1H | DRD1 | HEPACAM | NF1 | SCN2A | UPF3B |
| CACNA1I | DRD2 | HTR1A | NIPBL | SETD5 | VPS13B |
| CACNA2D3 | DRD3 | HTR1B | NLGN3 | SHANK2 | VPS14 |
| CACNB2 | DRD4 | HTR2A | NLGN4X | SHANK3 | WFS1 |
| CAMK2A | DRD5 | HTR2C | NOS1 | SLC25A12 | ZBTB20 |
| CDKL5 | DSCAM | IGSF9B | NPAS2 | SLC39A8 | ZNF536 |
| CHD2 | DTNBP1 | IMMP2L | NR1D1 | SLC6A2 | ZNF804A |
| CHD7 | DUSP6 | ITGB3 | NR3C1 | SLC6A3 | ZSWIM6 |
| CHD8 | DYRK1A | KATNAL2 | NRG1 | SLC6A4 |  |
| CHRNA4 | EGFR | KCNJ10 | NRXN1 | SLC9A6 |  |
